# Reputation Management in Children on the Autism Spectrum

**DOI:** 10.1007/s10803-016-2923-1

**Published:** 2016-09-30

**Authors:** Eilidh Cage, Geoffrey Bird, Elizabeth Pellicano

**Affiliations:** 1 Centre for Research in Autism and Education (CRAE), Department of Psychology and Human Development, UCL Institute of Education, University College London, London, UK; 2 Department of Psychology, Royal Holloway, University of London, Egham, Surrey, TW20 0EX UK; 3 MRC Social, Genetic & Developmental Psychiatry Centre, Institute of Psychiatry, Kings College London, London, UK; 4 Institute of Cognitive Neuroscience, University College London, London, UK; 5 School of Psychology, University of Western Australia, Perth, Australia

**Keywords:** Autism, Reputation management, Theory of mind, Social motivation, Inhibitory control, Reciprocity

## Abstract

Being able to manage reputation is an important social skill, but it is unclear whether autistic children can manage reputation. This study investigated whether 33 autistic children matched to 33 typical children could implicitly or explicitly manage reputation. Further, we examined whether cognitive processes—theory of mind, social motivation, inhibitory control and reciprocity—contribute to reputation management. Results showed that neither group implicitly managed reputation, and there was no group difference in explicit reputation management. Results suggested different mechanisms contribute to reputation management in these groups—social motivation in typical children and reciprocity in autistic children. Explicit reputation management is achievable for autistic children, and there are individual differences in its relationship to underlying cognitive processes.

## Introduction

Reputation—how we are seen in the eyes of others—is a social construct, used to predict how others might act in the future (Leimgruber et al. 2012). Reputation concerns are widespread in typical individuals: they donate more to charity when observed (Izuma et al. 2010, 2011), behave in more prosocial ways when a pair of eyes are present (Bateson et al. 2013, 2006) and strive to keep up appearances on the Internet (Tennie et al. 2010). From an evolutionary perspective, reputation is valuable for cooperation: individuals with a reputation for being cooperative are more likely to be selected as partners in the future (Barclay and Willer 2007; Sylwester and Roberts 2010; Tomasello et al. 2012; Tomasello and Vaish 2013).

Less is known, however, about reputation management—the efforts made to maintain or obtain a certain reputation—in those with autism. Autism is a pervasive neurodevelopmental condition that affects the way autistic individuals experience the world around them. In particular, autistic^1^ individuals struggle with social aspects of everyday life (American Psychiatric Association (APA) 2013) including problems with making friends (Petrina et al. 2014) and understanding others’ thoughts (Baron-Cohen et al. 1985; Tager-Flusberg 2007). It could be argued that, given these pervasive social difficulties, autistic children would be unable to manage their reputation.

However, there is mixed evidence as to whether children with autism are concerned about their reputation. Chevallier, Molesworth and Happé (2012b) tested whether children with autism would flatter when informed that a drawing they had previously seen had been drawn by the experimenter. While typical children increased their rating of this drawing, purportedly to manage reputation, autistic children did not. Chevallier et al. (2012b) interpreted this result as evidence for a lack of reputation management in autism. Research into self-presentational skills, however, suggests that autistic children may have some preserved reputation management ability. Self-presentation (or ‘explicit reputation management’) is the ability to deliberately present oneself in a certain light (Banaji and Prentice 1994). In Begeer et al.’s (2008) study, autistic and typical children could win a prize by describing why they deserved to win the prize. Like typical children, autistic children used more positive self-statements compared to when they were asked to describe themselves. However, children with autism were less strategic in their self-descriptions. These findings were replicated by Scheeren, Begeer, Banerjee, Meerum Terwogt and Koot (2010), who also noted that children with autism found it harder to target their self-descriptions to specific audiences. Scheeren et al. (2010) suggested that autistic children may be less skilled at self-presentation due to a reduced propensity to exaggerate or make up facts to gain prizes. However, Scheeren et al. (2015) subsequently found no difference between autistic and typical children in strategic self-presentation.

It seems plausible from self-presentation research (Barbaro and Dissanayake 2007; Begeer et al. 2008; Scheeren et al. 2010, 2015) that autistic children may be able to manage their reputation when there is an incentive to do so. One possible reputational incentive for autistic children is friendships. Autistic children do desire friendships (Bauminger et al. 2003; Calder et al. 2013; Daniel and Billingsley 2010; Locke et al. 2010); however their friendships are often qualitatively different from those of typical children: in a review of 24 studies, Petrina et al. (2014) noted that autistic children perceived lower levels of companionship, intimacy and closeness compared to their typical peers. Further, there is great variation in the extent to which autistic children want social contact—while some report wanting many friends, others prefer to be alone (Calder et al. 2013). Autistic children who want to have friends may be more likely to consider their reputation.

Overall, these findings suggest that children with autism do not *implicitly* or *automatically* manage their reputation (Chevallier et al. 2012b), but that the ability to *explicitly* or *consciously* do so may be preserved (Scheeren et al. 2015). This suggestion corroborates research with autistic adults, which report a reduced propensity to implicitly manage reputation (Izuma et al. 2011) but an intact, albeit reduced, ability to do so when reputation was more explicitly at risk (Cage et al. 2013). The current study tested both implicit and explicit reputation management within the same group of children with autism and a matched group of typical children. To measure implicit reputation management, children completed one-shot dictator games once when observed and once when unobserved. We utilised the dictator game since it is thought to have good reliability (Thomae et al. 2012) and to be an excellent experimental means for testing social behaviour (Camerer and Fehr 2002). To measure explicit reputation management, children were given the opportunity to protect their reputation following allegedly poor performance in a game.

It is important to enhance our understanding of whether autistic children can be concerned for their reputation under different circumstances. If autistic children can show some concern for their reputation and an understanding of how to manage it, this would have implications for our understanding of social behaviour and social capacity in autism, and the potential for an ability which has previously been claimed not to be possible in autism (e.g. Chevallier et al. 2012b; Izuma et al. 2011).

## Individual Differences in Reputation Management

Recent research has shown, however, that reputation management is possible in some adolescents and adults on the autism spectrum (Cage et al. 2013, 2016; Scheeren et al. 2015), which raises questions about the underlying abilities that might explain variability in explicit and implicit reputation management in autistic individuals. Two main hypotheses have been proposed to explain why autistic individuals have difficulties with reputation management. The first, the Theory of Mind (ToM) hypothesis (Baron-Cohen et al. 1985; Izuma et al. 2011), claims that autistic individuals’ social-cognitive difficulties are caused by a lack of ToM, the ability to interpret the thoughts and beliefs of others and the self (Baron-Cohen et al. 1985). If autistic children have difficulty in representing other minds, they would be unable to represent how they are viewed in the eyes of others, and thus be incapable of reputation management (Izuma et al. 2011). However, children with autism do not categorically fail ToM tests, although this may be dependent on verbal ability and age (Scheeren et al. 2013) and the use of alternative strategies (Begeer et al. 2010; Lind and Bowler 2009). Thus, individual differences in ToM ability may contribute to the extent to which autistic children manage reputation.

A second explanation for reduced reputation management is that autistic individuals are not socially motivated, which leads to difficulties in developing appropriate social-cognitive skills—including being able to manage reputation (Chevallier et al. 2012a, b). Yet, social motivation is not completely absent in those on the autism spectrum: many report a need for friendships (Bauminger et al. 2003; Calder et al. 2013; Locke et al. 2010) and desire to fit in with others (Carrington et al. 2003a, b; Daniel and Billingsley 2010; Portway and Johnson 2003). It is possible that individual variation in social motivation could contribute to autistic children’s tendency to manage reputation.

Two novel accounts of reputation management were also examined in the current study: reciprocity and inhibitory control. Reciprocity is a behavioural response contingent on another’s actions (Falk and Fischbacher 2006). Typical individuals highly value reciprocity (Kahneman 2003), with those who are more reciprocal seen as more cooperative and with a better reputation (Hoffman et al. 1998; Milinski et al. 2002; Molleman et al. 2013; Nowak et al. 2005). Understanding the principles of reciprocity and having expectations that others will reciprocate with you could underlie the ability to manage reputation. A lack of social reciprocity is a hallmark feature of autism, with autistic individuals demonstrating a reduced number of appropriate reciprocal social responses such as conversational turn-taking (APA 2013). Expectations of reciprocity from others are likely based on experiences of reciprocity (Hoffman et al. 1994, 1996, 2008). In terms of social experiences, typical children often do not tend to reciprocate autistic children’s friendships (Rotheram-Fuller et al. 2010) and may frequently neglect and ignore autistic children in the playground (Kasari et al. 2011). These experiences may lend themselves to a reduced expectation of reciprocity in autistic children, which has previously been noted in autistic adults (Cage et al. 2013).

The second unexamined factor that may contribute to variability in reputation management in autism is inhibitory control. Reputation management, by definition, requires strategic control of behaviour in order to further one’s reputation. Thus, on occasion, reputation management may require the inhibition of behaviours likely to impact negatively upon one’s reputation and the selection of behaviours with a more positive impact (Von Hippel and Gonsalkorale 2005). In support of the proposed link between reputation management and response inhibition are findings demonstrating that inhibitory control is an important predictor of social-emotional competence: for example, children with better inhibition skills are rated as more socially skilled (Rhoades et al. 2009) and show fewer externalizing behaviour problems in later childhood (Olson et al. 1999). There is mixed evidence as to whether children with autism have difficulties with inhibition (Christ et al. 2007; Corbett et al. 2009; Hill 2004b) making this a worthy topic of investigation. Accordingly, individual differences in inhibitory control in autism could contribute to variability in reputation management.

The relationships between inhibitory control, reciprocity and reputation management have not previously been examined in autistic children, and these factors in addition to social motivation and theory of mind were assessed to determine the extent, if any, they were related individual differences in implicit and explicit reputation management.

## Method

### Participants

Sixty-six cognitively-able children aged from 7 to 14 years took part in the current study (Table 1). Typical children (*n* = 33) were matched to autistic children (*n* = 33) on chronological age, *t*(64) = 1.42, *p* = .16, *r* = .17, and verbal mental age, *t*(64) = 0.37, *p* = .71, *r* = .05. Children were matched on verbal mental age, as measured by the Wechsler Abbreviated Scales of Intelligence—Second edition (WASI-II; Wechsler 2011), since language ability was required for many of the tasks. An additional six autistic children whose verbal mental age was more than 3 years lower than their chronological age were excluded from the sample. This cut-off was used since none of the typical children showed a discrepancy between chronological and verbal mental age of this magnitude. Although the typical group had a higher proportion of girls than the autism group, chi square confirmed that this difference was not significant, χ²(1) = 2.75, *p* = .097, φ =0.20.

**Table 1:** Descriptive statistics for chronological age, verbal mental age, social communication questionnaire (SCQ) score and autism diagnostic observation schedule—2nd edition (ADOS-2) scores

	Group		*p* value
	Typical	Autism	
N	33	33	
Gender (M:F)	21:12	27:6	0.097
Chronological age (in years)			
M (SD)	10.24 (2.00)	10.96 (2.11)	0.16
Range	6.92–14.21	7.18–14.32	
Verbal mental age (in years)			
M (SD)	10.52 (2.28)	10.31 (2.35)	0.71
Range	5.74–14.50	6.15–15.31	
SCQ			
M (SD)	4.67 (3.63)	23.88 (6.62)	<0.001
Range	0–14	11–38	
ADOS overall score			
M (SD)	–	10.62 (3.33)	
Range	–	7–19	

Verbal mental age calculated by dividing chronological age by 100 and multiplying this by verbal IQ score as measured by the WASI-II (Wechsler 2011). Cut off scores for the SCQ and ADOS are 15 and 7 respectively, with higher scores reflecting greater autism symptomatology

Typical children were recruited through schools and extra-curricular clubs in the greater London area. Autistic children were recruited through autism resource provisions attached to mainstream schools in London and community contacts. All autistic children had an independent clinical diagnosis of an autism spectrum condition (ASC) and scored above the cut-off scores of 7 for an ASC on the Autism Diagnostic Observation Schedule—2nd edition (ADOS-2; Lord et al. 2000) and of 15 on the Social Communication Questionnaire (SCQ) (Rutter et al. 2003). One child with autism fell below the cut-off on the SCQ but was retained in analyses since he had a clinical diagnosis of Asperger’s syndrome, met criteria on the ADOS, and his removal from the sample did not alter the results. Parents of typical (*n* = 27) children also completed the SCQ (Rutter et al. 2003); none showed elevated levels of autistic symptomatology.

All procedures performed in this study were in accordance with the ethical standards of the first and third author’s institutional research committee and with the 1964 Helsinki declaration and its later amendments or comparable ethical standards. Informed written consent was obtained from all parents and verbal consent from children included in the study.

### Design

The current study had a between-participants design, with the independent variable of group (autism or typical). The dependent variables for the explicit and implicit tasks, as well as the four proposed mechanisms, are outlined below.

### Materials and Procedure

All of the tasks took place within the context of an online gaming world called “Verden”. This context provided an overarching theme for the research that was developmentally appropriate for children aged 7 to 14 years. All tasks were presented on 13″ Windows 7 Toshiba Portege laptops using MATLAB (The Mathworks, Massachusetts, USA) and Cogent toolbox (LON, FIL, & ICN, London, UK).

#### Implicit Reputation Management Task

**Fig. 1:**
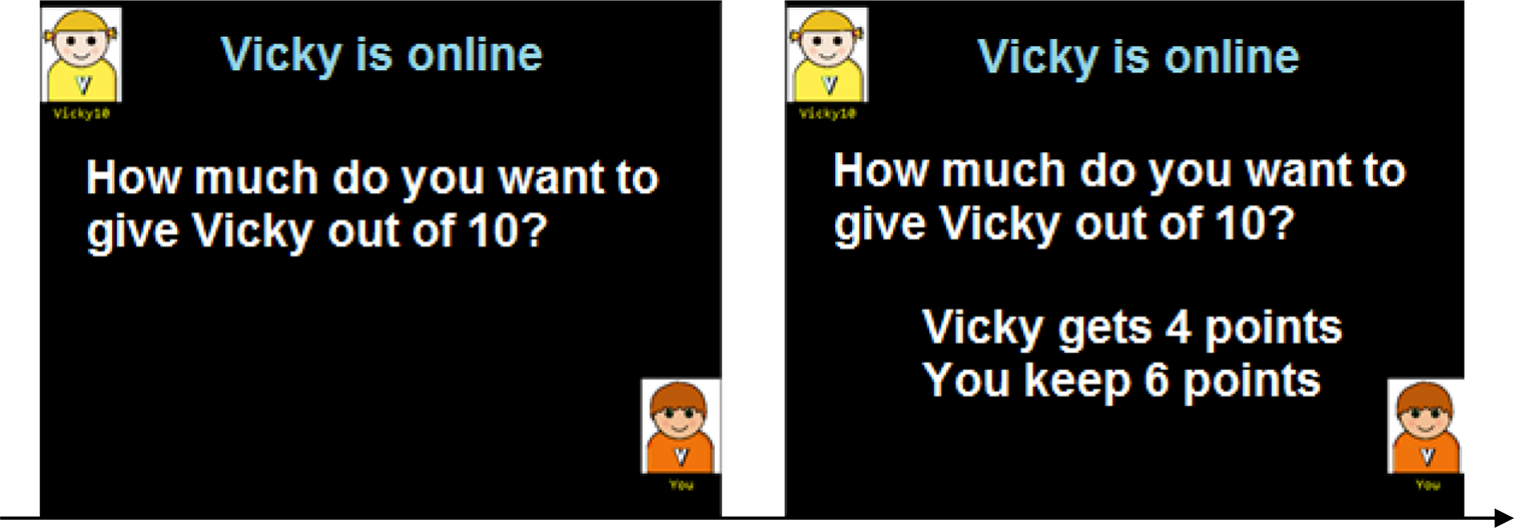
Example of a single trial from the implicit reputation task, in which children are first asked how many points they would like to share with the other player. After making their choice, the allocated points to the other and to the self were shown on-screen

This measure taps implicit reputation management by testing whether children are affected by the presence of an observer, by increasing the number of points given in the observed condition in order to appear more generous. In this task, children completed 20 one-shot dictator games: 10 when observed and 10 when unobserved. Children were instructed that they were going to play a decision making game: *“You are going to meet some of the other players in Verden. Each time you meet a new player, you will get 10 points. You can give him or her some or all of these points and you keep the rest”*. They were then asked, *“How much do you want to give [name of other player]?”* The number of points (between 0 and 10) was inputted using the keyboard (Fig. 1).

All children completed this task under two conditions: once unobserved and once whilst observed by another child. The other child was tested separately and concurrently on the same tasks with a second experimenter. To justify why the children needed to observe one another, an “error” occurred on one of the children’s laptops. The children then came together to complete the task on the “working” laptop. Once one child had completed the task observed, they exchanged places so that the other child could complete 10 observed trials. To ensure observation, the observing child was asked to write down the participating child’s responses. Once both had observed each other, the experimenter returned and claimed the broken laptop was fixed. If the unobserved condition was first, they moved on to the next task. If the observed condition was first, they completed the task again unobserved. The order of observed and unobserved conditions was counterbalanced across children. The dependent variable was the observer effect, obtained by calculating the difference score between observed and unobserved conditions.

#### Explicit Reputation Management Task

**Fig. 2:**
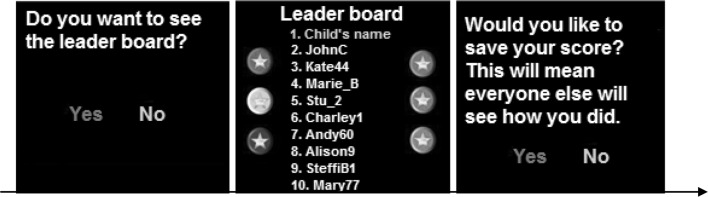
Example trial structure during the explicit reputation task. Children were first given the option to see the leader board, and if they decided to do so, they saw their position on the leader board (either first or eighth). They were then asked if they would like to save their score

This task was designed to give children the opportunity to prevent others knowing about poor performance in a game and to protect their reputation. This task was designed to have lower verbal demands than previous tasks testing self-presentation in autism (e.g. Barbaro and Dissanayake 2007; Begeer et al. 2008; Scheeren et al. 2010, 2015). Children were asked to test three computer games run through MATLAB. After playing each game, children were informed that other people in Verden had been playing the game, and a leader board was available. They were asked if they would like to view their position on the leader board. Unbeknownst to them, their position on the leader board was manipulated, such that they either came in first place or in eighth place (out of 10 players) on the leader board. Leader board position was counterbalanced. If children decided to view the leader board, they were asked whether they would like to save their position by making a yes/no judgment. It was emphasised that saving would mean that others would be able to view their position on the leader board (Fig. 2). Thus, the dependent variable was decision to save (yes or no).

#### Theory of Mind (ToM)

ToM was measured using White et al.’s (2009) version of the Strange Stories task (Happé 1994). Children saw six mental state stories and six nature stories from White et al.’s (2009) battery on-screen. Mental state stories are designed to measure mental state understanding while nature stories test general story comprehension (White et al. 2009). The experimenter read each story aloud and asked the child one question related to each story. Answers were scored 0 for an incorrect answer, 1 for a partially correct answer and 2 for a fully correct answer (maximum score 12 points for each story type, mental state or nature). The dependent variables were mean scores on mental state and nature stories; higher scores reflect better mental state and story comprehension, respectively.

#### Social Motivation

Social motivation was measured in two ways. In the first, ecologically-valid way, children were told that their assistance was required to test some new games in Verden. Critically, they had the choice of playing a two-player game (with the other child or the experimenter) or a one-player game (on their own).The dependent variable was the binary choice of playing with someone or playing alone.

Second, social motivation was measured using Richard and Schneider’s (2005) Friendship Motivation Questionnaire, which quantified children’s desire to be social through their motivation to have friends. Children were asked to think about why they wanted to have friends. They viewed 12 statements pertaining to the motivations for having friends; such as “*to be invited to parties”*, and “*because it makes me feel better when I’m sad”*. Children rated each statement on a 4-point scale by deciding how much the statement sounded like them from *not at all like me* (score of ‘1’) to *exactly like me* (score of ‘4’). A friendship motivation score was calculated by summing the responses on the scale. Higher scores indicate higher motivation for friendships (maximum score = 48).

#### Reciprocity: Baseline (Predictions of Generosity)

**Fig. 3:**
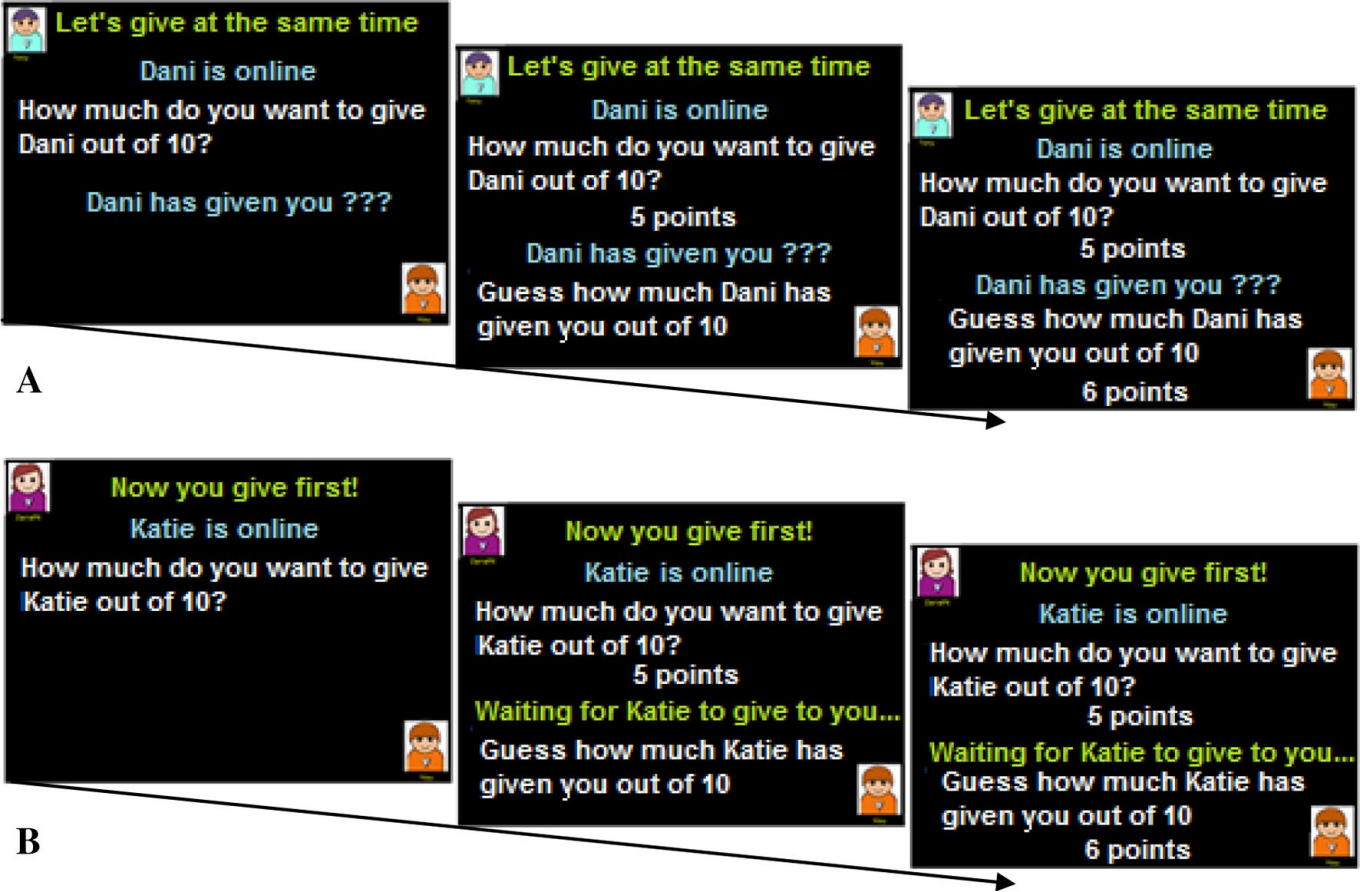
**a** Example trial structure of predictions of generosity. Children first give points to the other player, and then have to guess how many points the other has given to them. **b** Example trial structure of expectations of reciprocity. Children first decided how many points to give the other player, and then had to guess how many points the other would give to them

This task was designed to provide a baseline measure of children’s predictions of generosity from others. As with the implicit reputation management task, children were told that they were playing decision-making games. First, they had to decide how many points to give the other player *at the exact same time* as the other player decided how many points to give to them (Fig. 3a). Thus, there was no reciprocal element. After deciding how many points to give to the other player, children could next obtain ‘bonus points’ for guessing how many points the other would give them. No feedback was given regarding whether they had guessed correctly, but all children received a fixed amount of ‘bonus points’ at the end in order to ensure they stayed motivated by the task. The dependent variables of interest were the mean number of points children offered and guessed the other would give them (maximum 10 points). All children completed 10 trials, with one practice trial at the start.

#### Expectations of Reciprocity

This task followed a similar structure to the baseline condition, but children were informed that they would *give first* to the other player, and were then asked to guess how much the other player would give them in return (Fig. 3b). The experimenter informed the child that the other player would find out how many points they had been given prior to making their decision. This task consisted of one practice trial and 10 experimental trials, and the dependent variables were the mean number of points offered and guessed. This task examined whether children were aware that the other’s response could be contingent upon their own offer.

#### Inhibitory Control

Inhibitory control was tested with a go/no-go task. Following Cragg and Nation (2008), the go/no-go task was presented in the context of a football game. Children had to press the spacebar to “kick” a football every time it appeared on-screen. They were instructed to press the spacebar as fast as they could. Ten practice trials served to build a prepotent response to the football. Next, children were informed that they should continue to kick the footballs but *not* kick any rugby balls that appeared. Children completed two blocks of 50 trials each (100 trials in total), including 13 rugby balls within each block (26 %). The football or rugby ball appeared for 200 ms, with a random ISI between 1600 and 2600 ms between stimuli to ensure they could not predict when the stimulus would appear. The dependent variable was the child’s mean d’ score calculated across the two blocks. d’ was calculated by taking into account the number of trials in which children had correctly kicked the football (hit rate) and incorrectly kicked the rugby ball (false alarm rate). Higher d’ scores reflect better inhibitory control.

## Results

### Examining Between-Group Differences

#### Implicit Reputation Task

**Fig. 4:**
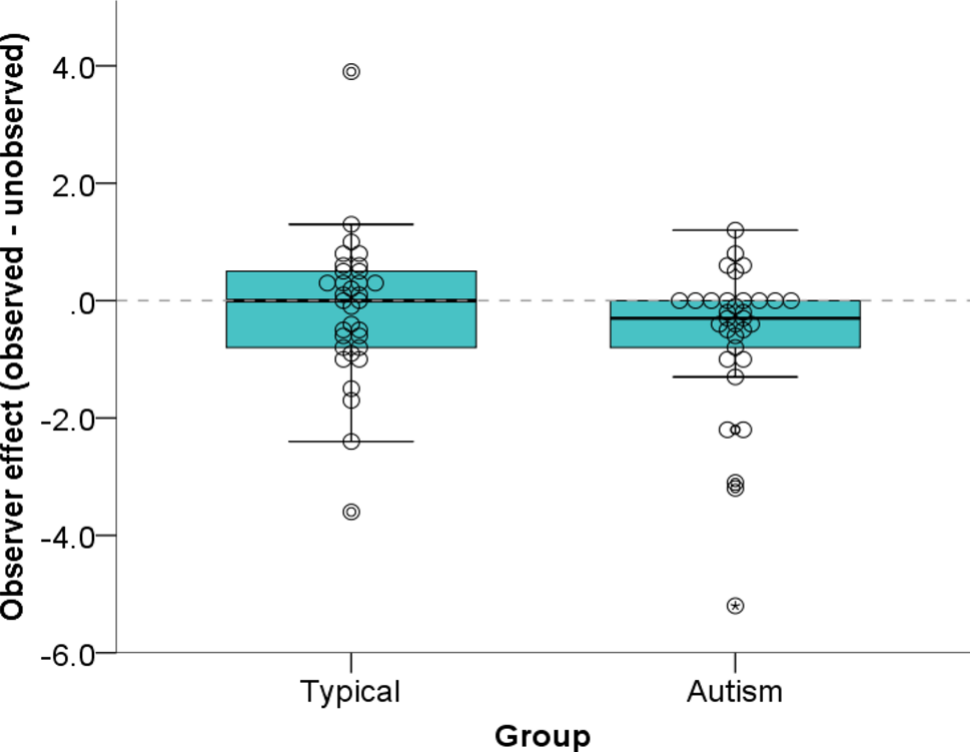
Box plots showing the distribution of the observer effect (the difference score between observed and unobserved conditions), for both typical and autism groups. The dotted line represents no difference between being observed and unobserved (i.e. no observer effect). Positive values are indicative of an observer effect

The observer effect, which quantifies the effect of being watched, can be seen in Fig. 4. A one-way between-participants ANOVA showed that there was no significant group difference, *F*(1, 64) = 2.24, *p* = .14, η_p_^2^ = 0.034.

**Fig. 5:**
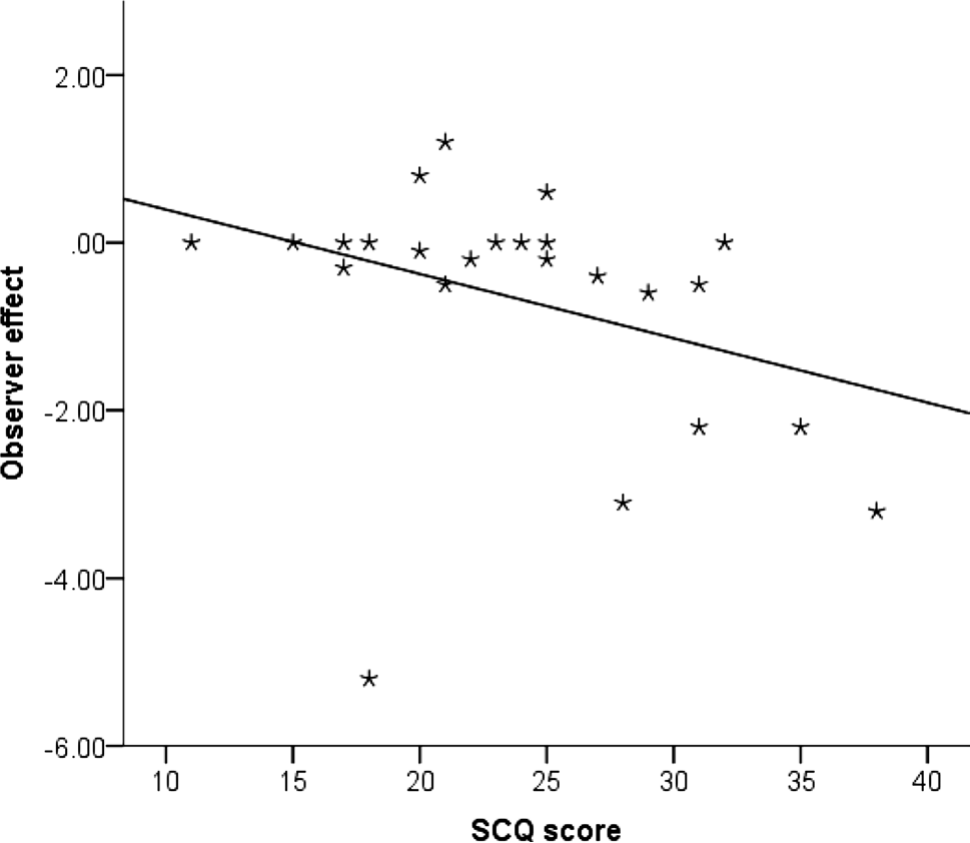
The relationship between the observer effect and score on the Social Communication Questionnaire (SCQ) in the autism group

One sample *t* tests were used to test whether the observer effect was significantly different from zero, which would indicate a change in behaviour when observed. For autistic children, there was a significant difference from zero, t(32) = −.2.73, *p* = .01, *r* = .43, but, as shown in Fig. 4, this effect is a negative response to observation. There was no significant difference from zero for the typical group, t(32) = −0.67, *p* = .51, *r* = .12. Exploratory analyses were conducted to examine this result. For children with autism, correlational analyses revealed a significant correlation between the observer effect and scores on the Social Communication Questionnaire (SCQ), *r*(31) = −0.35, *p* = .048, 1-tailed. We had a directional hypothesis that with increasing symptom severity, the observer effect would decrease (Fig. 5).

#### Explicit Reputation Task

In this task, children had the opportunity to protect their reputation. The number of children in each group deciding to save their position on the leader board, when either placed top or near the bottom, is shown in Table 2. Some children from each group chose not to see the leader board at all: when top, one typical child and three autistic children opted not to view the leader board. When bottom, one typical and two autistic children decided not to view the leader board.

**Table 2:** Number of children deciding to save or not to save their leader board position depending on whether they appeared top or bottom of the leader board

	Position			
	Top of leader board		Bottom of leader board	
Save?	Yes	No	Yes	No
Typical	31	1	12	20
Autism	28	2	17	14

Considering decisions when top of the leader board, the majority of typical children (96.9 %) and autistic children (93.3 %) wanted to save their position. Binomial tests showed that both groups were significantly above chance for saving when top of the leader board (both *p*s < 0.001). Fisher’s Exact Test showed no association between group and decision to save when top of the leader board, *p* = .61. When bottom of the leader board, 62.5 % of typical children and 45.2 % of autistic children did *not* want to save their position. Binomial tests revealed that both groups showed no distinct preference for whether they saved their score when bottom of the leader board (both *p*s > 0.22). Chi square analysis showed no significant association between group and decision to save, *χ²*(1) = 1.91, *p* = .18, φ = 0.17.

#### Theory of Mind

A 2 (group: typical or autism) × 2 (story type: mental state and nature) mixed ANOVA was conducted on Strange Stories task scores (Table 3). There was a significant main effect of group, *F*(1, 64) = 6.71, *p* = .012, η_p_^2^ = 0.095, with children with autism scoring significantly lower on both mental state and nature stories. All other main effects and interactions were not significant (*p*s > 0.41). An ANCOVA controlling for verbal mental age and gender did not change these results, although there was a significant main effect of verbal mental age, *F*(1, 62) = 32.52, *p* < .001, η_p_^2^ = 0.34.

**Table 3:** Mean (standard deviation) results for the theory of mind, social motivation, understanding and expectations of reciprocity, and inhibitory control tasks for autistic and typical children

Measure	Autism (n = 33)	Typical (n = 33)	p value
Theory of mind			
Mental state stories			
M (SD)	6.79 (3.19)	8.58 (1.94)	0.008
Range	0–11	2–12	
Nature stories			
M (SD)	6.88 (3.04)	8.12 (2.43)	0.059
Range	1–11	2–12	
Social motivation			
Friendship motivation score			
M (SD)	37.03 (5.38)	35.82 (4.81)	0.34
Range	21–44	23–44	
Inhibitory control			
Hit rate			
M (SD)	92.3 % (8.24 %)	93.9 % (6.59 %)	0.75
Range	61–100 %	80–100 %	
False alarm rate			
M (SD)	39.6 % (21.1 %)	34.9 % (19.6 %)	0.35
Range	4–92 %	8–85 %	
d′			
M (SD)	1.89 (0.95)	2.03 (0.89)	0.53
Range	0.08–3.87	−0.27–3.73	

#### Social Motivation

Social motivation was measured by asking children whether they would like to play a game with someone or alone. The majority of children in each group (typical 61 %, autism 64 %) preferred to play with someone. Chi square confirmed that there were no group difference, χ²(1) = 0.80, *p* = .80, φ = 0.11.

The Friendship Motivation Questionnaire (Richard and Schneider 2005) was also used as a measure of social motivation (Table 3). There was no significant difference between autistic and typical children in their motivation for friendships, *t*(64) = 0.96, *p* = .34, *r* = .12.

#### Reciprocity

**Fig. 6:**
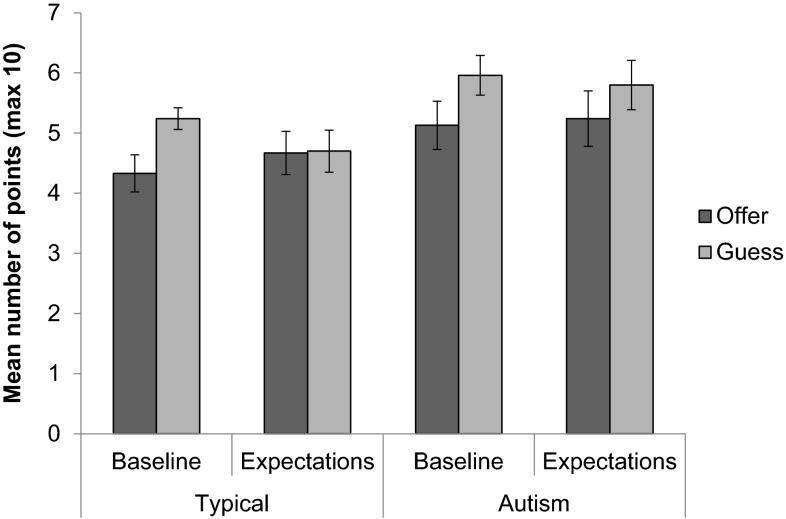
Mean number of points (maximum 10) offered and guessed by both groups according to whether the child was giving at the same time (baseline) or giving first (expectations of reciprocity). *Error bars* indicate ±one standard error of the mean

Children’s expectations of reciprocity were tested by giving them the opportunity to offer points to others and to guess how many points the other would give them under two conditions—a baseline condition (predictions of generosity) and an expectation of reciprocity condition. Figure 6 displays the mean number of points offered and guessed in each of these conditions.

A 2 (group: typical or autism) × 2 (condition: baseline and expectations of reciprocity) × 2 (decision: offer and guess) mixed ANOVA revealed a significant main effect of decision, *F*(1, 63) = 6.46, *p* = .014, η_p_^2^ = 0.093, such that the number of points children guessed the other player would give them was higher than the number of points they offered. There was a significant condition x decision interaction, *F*(1, 63) = 5.25, *p* = .025, η_p_^2^ = 0.077 and the main effect of group approached significance, *F*(1, 63) = 3.66, *p* = .06, η_p_^2^ = 0.06, but there were no other significant main effects or interactions (all *p*s > 0.23). To examine the interaction between condition and decision, we conducted follow-up analyses using repeated-measures *t* tests. These analyses showed only a difference approaching significance between *guesses* at baseline and the expectation of reciprocity condition, *t*(64) = 1.95, *p* = .055, *r* = .24, such that guesses were higher at baseline. An additional ANCOVA controlling for verbal mental age (since verbal ability could affect understanding of reciprocity) revealed a significant main effect of group, *F*(1, 61) = 4.02, *p* = .05, η_p_^2^ = 0.06, and verbal mental age, *F*(1, 61) = 5.87, *p* = .018, η_p_^2^ = 0.09, such that autistic children tended to both offer and guess more points than typical children, and that overall variability could be explained by verbal ability.

#### Inhibitory Control

A MANOVA on the hit and false alarm rates (Table 3) showed that there were no significant differences between groups in hit rate, *F*(1, 64) = 0.10, *p* = .75, η_p_^2^ = 0.002, or false alarm rate, *F*(1, 64) = 0.86, *p* = .34, η_p_^2^ = 0.013. There were also no significant difference between groups on d’ scores, *t*(64) = 0.63, *p* = .53, *r* = .08.

## Examining Individual Differences: Relationships Between Mechanisms and Reputation Management

We tested whether there were any relationships between performance on the tasks tapping putative mechanisms and reputation management in autism and typical children by using correlations and partial correlations (controlling for verbal mental age) within each group separately. The results of these analyses revealed few significant correlations. The exceptions were significant correlations between autistic children’s verbal mental age and ToM scores (*r*(31) = 0.64, *p* < .001). For typical children, partial correlations between ToM and friendship motivation were significant (*r* (31) = 0.47, *p* = .008) as was the correlation between explicit reputation management task and friendship motivation (*r* (31) = 0.40, *p* = .03).

Further exploratory analyses were conducted since explicit reputation management had a binary response as a dependent variable. We tested whether there were any differences between children who had decided to say “yes” or “no” to saving their position when bottom of the leader board. There were only significant differences within the reciprocity tasks for autistic children (Table 4). Autistic children who said “yes” to saving when bottom of the leader board (those who did *not* protect their reputation) made higher offers in the baseline reciprocity condition, *t*(29) = 3.17, *p* = .004, *r* = .51, and made higher offers in the expectations of reciprocity condition, *t*(29) = 2.18, *p* = .037, *r* = .38.

**Table 4:** Descriptive statistics for measures of reciprocity which significantly differ between autistic children who said “yes” or “no” to saving when they came bottom of the leader board

	Save when bottom of the leader board?	
Measure	Yes	No
Offer when giving at the same time	6.12 (1.99)*	4.66 (1.67)
Offer when giving first	6.65 (2.31)**	4.26 (1.78)

*p < .05, **p < .01 for offers significantly different to 5 points

One-sample *t* tests tested whether autistic children differed significantly from an offer of 5 points, which could be considered a fair offer. Autistic children who had said “yes” to saving when bottom of the leader board (those less concerned about their reputation) made offers significantly higher than a fair offer in the baseline condition, *t*(16) = 2.32, *p* = .034, *r* = .50, and in the expectations of reciprocity condition, *t*(16) = 2.94, *p* = .01, *r* = .59. There was no difference from a fair offer for autistic children who had said “no” to saving when bottom of the leader board. These results suggest that autistic children who protected their reputation were fairer during the reciprocity tasks.

## Discussion

The current study examined whether autistic children could implicitly or explicitly manage their reputation and the potential processes underpinning such abilities. As predicted, children with autism did not implicitly manage their reputation, and in fact showed a reverse observer effect in that they gave *fewer* points to an anonymous other when observed. When reputation was more explicitly at risk, some—but not all—autistic children decided to protect their reputation. Typical children did not manage their reputation in an implicit situation, and there was no difference between the groups in terms of explicit reputation management.

Our results partially support previous research testing implicit reputation management in autistic children (Chevallier et al. 2012b). The methodology of Chevallier et al.’s (2012b) study, however, differed markedly to that of the current study. Children in their study demonstrated reputation management by increasing ratings of a drawing that the experimenter claimed that she had drawn herself. In the current study, children were observed by a third party whilst playing dictator games in an online gaming world. Interestingly, typical children were not sensitive to observation in our study. This may be due to a protracted development of this ability, which may not emerge until adolescence (Blakemore and Mills 2014) and can be more clearly seen in adulthood (e.g. Bateson et al. 2006). Results showed that autistic children in fact gave fewer points when observed, and this was correlated with their SCQ scores such that with those with the greatest degree of autistic features showed weaker observer effects. Perhaps autistic children with greater symptom severity may have found observation more aversive and thus changed their behaviour. This result is in line with previous research suggesting that atypical reactions to eye gaze can be related to more severe symptomatology (Leekam et al. 1998; Klin et al. 2002).

In the explicit reputation task, in which children had the opportunity to protect their reputation, there was a tendency for some autistic children to protect their reputation. These findings are consistent with research conducted on autistic children’s self-presentation skills, which suggests the ability to present oneself in a certain way is intact (Begeer et al. 2008; Scheeren et al. 2010, 2015). It appears that *some* autistic children can be aware when their reputation is explicitly at stake and they can take a simple step of preventing others knowing about this in a computer game. Real-life explicit reputation management is likely to be more complicated, and indeed Begeer et al. (2008) and Scheeren et al. (2010) note that while autistic children can present themselves in a certain light, they do so with less skill—although they did not replicate this finding in a larger sample (Scheeren et al. 2015). Nonetheless it is important to consider this gap between knowledge of reputation and reputation management in action, supporting the idea that autistic individuals may be aware of reputation, but struggle with the social skills needed to effectively manage it, a finding noted previously in autistic adults (Cage et al. 2013).

We also tested whether different cognitive processes might determine the degree to which children with autism engage in reputation management. Scores on tasks designed to measure individual differences in the candidate mechanisms, however, were unrelated to individual differences in implicit reputation management. Further analyses, however, revealed potentially interesting differences within the group of autistic children—between those who had protected their reputation and those who had not, offering potential explanations for why some but not all autistic children managed their reputation. Specifically, autistic children who protected their reputation made fairer offers in the reciprocity task. It may be the case that those with a more sensitive appreciation of fairness are also more sensitive to their reputation when it is explicitly at risk. This finding supports previous research, which has suggested that understanding and expectations of reciprocity may contribute to reputation management in autism (Cage et al. 2013). Further, our results suggest that autistic children, like their typical peers, may use fairness as a signal to others (Shaw 2013; Shaw et al. 2014). Fairness is an important motivator of behaviour, especially in economic games (Fehr and Gächter 2000; Fehr and Schmidt 1999) and the current findings support research which demonstrates that autistic children have explicit awareness of social norms such as equality or fairness (Schmitz et al. 2014). Indeed, Scheeren et al. (2010) claim that autistic individuals may be less effective in self-presentation due to an increased likelihood of sticking to norms—and thus they avoid self-presentational techniques such as lying or boasting to boost their reputation. Thus, learning about norms and social rules may contribute to variability in explicit reputation management in autism. However, caution is warranted in the interpretation of this result since these analyses were post-hoc in nature. Nevertheless, they do highlight interesting hypotheses for future research. Such research should also consider other alternative explanations for why not all autistic children explicitly manage reputation—such as the impact of social anxiety.

Conversely, we found that for typical children, social motivation was related to explicit reputation management, such that those who reported a higher friendship motivation score were more likely to protect their reputation. This mechanism did not impact upon reputation management for autistic children. These findings suggest that how autistic children come to manage their reputation is likely to be different to how typical children manage reputation. Although some of the candidate mechanisms did not relate to reputation management, there were some interesting group differences in these tasks, which are outlined below.

Our findings concerning social motivation contradict the social motivation hypothesis (Chevallier et al. 2012a). Children with autism in the current study chose to play with someone rather than alone just as much as typical children and they expressed a similar degree of motivation for friendships on a questionnaire measure (Richard and Schneider 2005). These results indicate that autistic children *can* be socially motivated, supporting other research that suggests that this is the case (Calder et al. 2013; Deckers et al. 2014; Ewing et al. 2013; Locke et al. 2010). Second, previous studies have found mixed and inconsistent results on inhibitory control in autism (Christ et al. 2007; Hill 2004a, b; Ozonoff and Strayer 1997), and the current study found no difference in performance between autistic children and typical children in the go/no-go task, supporting the claim that autistic individuals do not have difficulties with response inhibition (Adams and Jarrold 2012). It is worth noting that the go/no-go task requires a number of abilities including response selection, response inhibition and decision-making (Rubia et al. 2001), thus an alternative task, such as the stop-signal task, may be more appropriate in future research (Lipszyc and Schachar 2010).

Notably, there was a significant group difference on the Strange Stories task (Happé 1994; White et al. 2009) but this group difference was not specific to mental state understanding, as performance on nature stories was also significantly poorer than typical children. Performance on the Strange Stories task was significantly related to verbal ability, as expected (Happé 1995; Scheeren et al. 2013). These results suggest that their performance was more contingent on verbal ability and story comprehension rather than a specific difficulty with ToM. Regardless of these data, any difficulties in ToM in autism may be insufficient to explain the social difficulties found in autism (Bennett et al. 2013; Pellicano 2013). Indeed, in a review of interventions based on ToM there was little evidence that such interventions had an impact on real-life social skills (Fletcher-Watson et al. 2014). To examine further the relationship between reputation management and ToM, tasks examining both cognitive (perspective taking) and affective aspects (emotion understanding) (Sebastian et al. 2011) should be used. It may be the case that cognitive ToM contributes more to reputation concerns than affective ToM, given the suggestion that cognitive ToM relates more to understanding other’s beliefs (in this case, about one’s reputation) (Sebastian et al. 2011).

Whether we expect others to reciprocate could be an important mediator for decisions related to reputation management, for example, when deciding to trust someone (Tanis and Postmes 2005). Initial analyses suggested that there were no differences in expectations of reciprocity between autistic and typical children, with both groups tending to have high predictions of generosity (such that they guessed the other would give them more points than they themselves were prepared to give) and then adjusting this when the possibility of reciprocity was introduced, suggesting that they were aware that the other’s response would be contingent on their own offer. However, after controlling for verbal ability, autistic children gave significantly more points overall, and verbal mental age accounted for a significant amount of variance in the number of points given, suggesting that verbal ability may impact on expectations of reciprocity. It may be the case that those with better verbal ability are more adept at understanding social norms (such as reciprocity), which are thought to have evolved precisely because of language (Smith 2010), and thus expectations could be related to the knowledge and experience of these norms (Hoffman et al. 2008).

### Limitations and Future Directions

This study is not without its limitations. It is possible that the tasks used were not sufficiently sensitive to detect potential differences between autistic and typical children. The Strange Stories task (Happé 1994) is frequently used to measure second-order ToM (e.g. White et al. 2009), yet was only related to differences in children’s language ability. The go/no-go task has also previously found mixed results (Adams and Jarrold 2012; Christ et al. 2007; Hill 2004a). Previous research utilising economic games to test social decision-making in autistic children have also shown little difference between typical and autistic children (Downs and Smith 2004; Sally and Hill 2006), although recent research suggests that children with autism may have different norm preferences (Schmitz et al. 2014). Therefore, one would expect there to be individual differences within the various tasks, which indeed we found. However, only variability in friendship motivation contributed to explicit reputation management, and only in typical children. With regard to the sensitivity of our implicit reputation management task, again, we found great individual differences within this task. Similar implicit tasks have been used in adult populations (Cage et al. 2013; Izuma et al. 2011) suggesting that the task is valid, but over the course of development children may become more aware that their reputation at stake in this task. Thus, future research would benefit from testing implicit reputation management in adolescents to examine its developmental trajectory.

Understanding autistic individual’s concern for reputation has implications for how autism is viewed—some autistic individuals can be concerned about how they are seen in the eyes of others and we should take this into consideration, for example in the classroom. This suggestion also corroborates research demonstrating social interest in autism (e.g. Calder et al. 2013; Deckers et al. 2014; Ewing et al. 2013; Locke et al. 2010). Our findings present further evidence for potential social capacity in autism. Future research should examine the development of this capacity in adolescence, especially since qualitative evidence suggests that autistic adolescents can be concerned about their reputation (Cage et al. 2016) and adolescence appears to be a pertinent time for reputation concerns in typical individuals (Blakemore and Mills 2014). Finally, although the current study did not find gender differences within any of the tasks, future research would benefit from a larger female sample to test for any potential gender differences, particularly given the suggestion that autistic girls may be better at “camouflaging” or “masking” (Head et al. 2014) and therefore cognizant of how others’ might perceive them.

Overall, the current study supported the hypothesis that some autistic children can manage their reputation explicitly, but not implicitly. Our results suggest that autistic children may be less susceptible to being automatically or subconsciously influenced by other people, but they are not immune to explicit awareness that their behaviour could be judged by others. However, there were individual differences in explicit reputation management in autism, with some, but not all, autistic children taking steps to influence what others know about them. The current findings also highlight that the ability to manage reputation explicitly may be underpinned by different mechanisms in typical and in autistic children. Further research is required to strengthen our understanding of reputation management in autism, and to examine alternative hypotheses.

## Footnotes

1 The term ‘autistic person’ is the preferred language of many people on the spectrum (e.g. Kenny et al. 2015; Sinclair 1999). In this article, we use this term as well as person-first language to respect the wishes of all individuals on the spectrum (Kenny et al. 2015).

## References

[CR1] Adams N. C., Jarrold C. (2012). Inhibition in autism: Children with autism have difficulty inhibiting irrelevant distractors but not prepotent responses. Journal of autism and developmental disorders.

[CR2] American Psychiatric Association (2013). Diagnostic and statistical manual of mental disorders.

[CR3] Banaji M. R., Prentice D. A. (1994). The self in social contexts. Annual Review of Psychology.

[CR4] Barbaro J., Dissanayake C. (2007). A comparative study of the use and understanding of self-presentational display rules in children with high functioning autism and asperger’s disorder. Journal of Autism and Developmental Disorders.

[CR5] Barclay P., Willer R. (2007). Partner choice creates competitive altruism in humans. Proceedings of the Royal Society B: Biological Sciences.

[CR6] Baron-Cohen S., Leslie A. M., Frith U. (1985). Does the autistic child have a theory of mind?. Cognition.

[CR7] Bateson M., Callow L., Holmes J. R., Redmond Roche M. L., Nettle D. (2013). Do images of “Watching Eyes” induce behaviour that is more pro-social or more normative? A field experiment on littering. PLoS One.

[CR8] Bateson M., Nettle D., Roberts G. (2006). Cues of being watched enhance cooperation in a real-world setting. Biology Letters.

[CR9] Bauminger N., Cory S., Agam G. (2003). Peer interaction and loneliness in high-functioning children with autism. Journal of Autism and Developmental Disorders.

[CR10] Begeer S., Banerjee R., Lunenburg P., Meerum Terwogt M., Stegge H., Rieffe C. (2008). Brief report: Self-presentation of children with autism spectrum disorders. Journal of Autism and Developmental Disorders.

[CR11] Begeer S., Malle B. F., Nieuwland M. S., Keysar B. (2010). Using Theory of Mind to represent and take part in social interactions: Comparing individuals with high-functioning autism and typically developing controls. European Journal of Developmental Psychology.

[CR12] Bennett T. A., Szatmari P., Bryson S., Duku E., Vaccarella L., Tuff L. (2013). Theory of mind, language and adaptive functioning in ASD: A neuroconstructivist perspective. Journal of the Canadian Academy of Child and Adolescent Psychiatry.

[CR13] Blakemore S.-J., Mills K. L. (2014). Is adolescence a sensitive period for sociocultural processing?. Annual Review of Psychology.

[CR14] Cage E., Bird G., Pellicano E. (2016). “I am who I am”: Reputation concerns in adolescents with autism. Research in Autism Spectrum Disorders.

[CR15] Cage E., Pellicano E., Shah P., Bird G. (2013). Reputation management: evidence for ability but reduced propensity in Autism. Autism Research.

[CR16] Calder L., Hill V., Pellicano E. (2013). “Sometimes I want to play by myself”: Understanding what friendship means to children with autism in mainstream primary schools. Autism: The International Journal of Research and Practice.

[CR17] Camerer C. F., Fehr E., Henrich J., Boyd R., Bowles S., Camerer C., Fehr E., Gintis H. (2002). Measuring social norms and preferences using experimental games: A guide for social scientists. Foundations of human sociality: Economic experiments and ethnographic evidence from fifteen small-scale societies.

[CR18] Carrington, S., Papinczak, T., & Templeton, E. (2003a). A phenomenological study: The social world of five adolescents who have Asperger’s syndrome. *Australian Journal of Learning Disabilities, 8*(3), 15–20.

[CR19] Carrington, S., Templeton, E., & Papinczak, T. (2003b). Adolescents with Asperger syndrome and perceptions of friendship. *Focus on Autism and Other Developmental Disabilities, 18*(4), 211–218.

[CR20] Chevallier, C., Kohls, G., Troiani, V., Brodkin, E. S., & Schultz, R. T. (2012a). The social motivation theory of autism. *Trends in Cognitive Sciences, 16*(4), 231–239. 10.1016/j.tics.2012.02.007 PMC3329932 22425667

[CR21] Chevallier, C., Molesworth, C., & Happé, F. (2012b). Diminished social motivation negatively impacts reputation management: Autism spectrum disorders as a case in point. *PLoS One, 7*(1), e31107. 10.1371/journal.pone.0031107 PMC3267764 22303483

[CR22] Christ S., Holt D., White D., Green L. (2007). Inhibitory control in children with autism spectrum disorder. Journal of Autism and Developmental Disorders.

[CR23] Corbett B. A., Constantine L. J., Hendren R., Rocke D., Ozonoff S. (2009). Examining executive functioning in children with autism spectrum disorder, attention deficit hyperactivity disorder and typical development. Psychiatry Research.

[CR24] Cragg L., Nation K. (2008). Go or no-go? Developmental improvements in the efficiency of response inhibition in mid-childhood. Developmental Science.

[CR25] Daniel L. S., Billingsley B. S. (2010). What boys with an autism spectrum disorder say about establishing and maintaining friendships. Focus on Autism and Other Developmental Disabilities.

[CR26] Deckers A., Roelofs J., Muris P., Rinck M. (2014). Desire for social interaction in children with autism spectrum disorders. Research in Autism Spectrum Disorders.

[CR27] Downs A., Smith T. (2004). Emotional understanding, cooperation, and social behavior in high-functioning children with autism. Journal of Autism and Developmental Disorders.

[CR86] Ewing, L., Pellicano, E., & Rhodes, G. (2013). Reevaluating the selectivity of face-processing difficulties in children and adolescents with autism. *Journal of Experimental Child Psychology, 115*(2), 342–355. 10.1016/j.jecp.2013.01.009 23563163

[CR28] Falk A., Fischbacher U. (2006). A theory of reciprocity. Games and Economic Behavior.

[CR29] Fehr E., Gächter S. (2000). Fairness and retaliation: The economics of reciprocity. The Journal of Economic Perspectives.

[CR30] Fehr E., Schmidt K. M. (1999). A theory of fairness, competition, and cooperation. The Quarterly Journal of Economics.

[CR31] Fletcher-Watson, S., McConnell, F., Manola, E., & McConachie, H. (2014). Interventions based on the theory of mind cognitive model for autism spectrum disorder. *Cochrane Database of Systematic Reviews* (3), Art. No.:CD008785. 10.1002/14651858.CD008785.pub2 PMC6923148 24652601

[CR32] Happé F. G. E. (1994). An advanced test of theory of mind - understanding of story characters thoughts and feelings by able autistic, mentally handicapped, and normal children and adults. Journal of Autism and Developmental Disorders.

[CR33] Happé, F. G. E. (1995). The role of age and verbal ability in the theory of mind task performance of subjects with autism. *Child Development, 66*(3), 843–855. 7789204

[CR34] Head A. M., McGillivray J. A., Stokes M. A. (2014). Gender differences in emotionality and sociability in children with autism spectrum disorders. Molecular Autism.

[CR35] Hill, E. L. (2004a). Evaluating the theory of executive dysfunction in autism. *Developmental Review, 24*(2), 189–233.

[CR36] Hill, E. L. (2004b). Executive dysfunction in autism. *Trends in Cognitive Sciences, 8*(1), 26–32. 10.1016/j.tics.2003.11.003 14697400

[CR37] Hoffman E., McCabe K., Shachat K., Smith V. (1994). Preferences, property rights, and anonymity in bargaining games. Games and Economic Behavior.

[CR38] Hoffman E., McCabe K., Smith V. (2008). Reciprocity in ultimatum and dictator games: An introduction. Handbook of Experimental Economics Results.

[CR39] Hoffman, E., McCabe, K., & Smith, V. L. (1996). Social distance and other-regarding behavior in dictator games. *The American Economic Review*, 653–660.

[CR40] Hoffman E., McCabe K. A., Smith V. L. (1998). Behavioral foundations of reciprocity: Experimental economics and evolutionary psychology. Economic Inquiry.

[CR41] Izuma K., Matsumoto K., Camerer C. F., Adolphs R. (2011). Insensitivity to social reputation in autism. Proceedings of the National Academy of Sciences.

[CR42] Izuma K., Saito D. N., Sadato N. (2010). Processing of the incentive for social approval in the ventral striatum during charitable donation. Journal of Cognitive Neuroscience.

[CR43] Kahneman D. (2003). A psychological perspective on economics. The American Economic Review.

[CR44] Kasari C., Locke J., Gulsrud A., Rotheram-Fuller E. (2011). Social networks and friendships at school: Comparing children with and without ASD. Journal of Autism and Developmental Disorders.

[CR45] Kenny, L., Hattersley, C., Molins, B., Buckley, C., Povey, C., & Pellicano, E. (2015). Which terms should be used to describe autism? Perspectives from the UK autism community. *Autism*, 1362361315588200. 10.1177/1362361315588200 26134030

[CR46] Klin A., Jones W., Schultz R., Volkmar F., Cohen D. (2002). Visual fixation patterns during viewing of naturalistic social situations as predictors of social competence in individuals with autism. Archives of General Psychiatry.

[CR47] Leekam S. R., Hunnisett E., Moore C. (1998). Targets and Cues: Gaze-following in Children with Autism. Journal of Child Psychology and Psychiatry.

[CR48] Leimgruber K. L., Shaw A., Santos L. R., Olson K. R. (2012). Young children are more generous when others are aware of their actions. PLoS ONE.

[CR49] Lind S. E., Bowler D. M. (2009). Language and theory of mind in autism spectrum disorder: the relationship between complement syntax and false belief task performance. Journal of Autism and Developmental Disorders.

[CR50] Lipszyc J., Schachar R. (2010). Inhibitory control and psychopathology: A meta-analysis of studies using the stop signal task. Journal of the International Neuropsychological Society.

[CR51] Locke J., Ishijima E. H., Kasari C., London N. (2010). Loneliness, friendship quality and the social networks of adolescents with high-functioning autism in an inclusive school setting. Journal of Research in Special Educational Needs.

[CR52] Lord C., Risi S., Lambrecht L., Cook E. H., Leventhal B. L., DiLavore P. C., Pickles A., Rutter M. (2000). The autism diagnostic observation schedule—generic: A standard measure of social and communication deficits associated with the spectrum of autism. Journal of Autism and Developmental Disorders.

[CR53] Milinski M., Semmann D., Krambeck H.-J. (2002). Reputation helps solve the ‘tragedy of the commons’. Nature.

[CR54] Molleman L., van den Broek E., Egas M. (2013). Personal experience and reputation interact in human decisions to help reciprocally. Proceedings of the Royal Society B: Biological Sciences.

[CR55] Nowak M. A., Page K. M., Sigmund K. (2000). Fairness versus reason in the ultimatum game. Science.

[CR56] Olson S. L., Schilling E. M., Bates J. E. (1999). Measurement of impulsivity: Construct coherence, longitudinal stability, and relationship with externalizing problems in middle childhood and adolescence. Journal of Abnormal Child Psychology.

[CR57] Ozonoff S., Strayer D. (1997). Inhibitory function in nonretarded children with autism. Journal of Autism and Developmental Disorders.

[CR58] Pellicano E. (2013). Testing the predictive power of cognitive atypicalities in autism: Evidence from a 3-year follow-up study. Autism Research.

[CR59] Petrina N., Carter M., Stephenson J. (2014). The nature of friendship in children with autism spectrum disorders: A systematic review. Research in Autism Spectrum Disorders.

[CR60] Portway S., Johnson B. (2003). Asperger syndrome and the children who don’t quite Fit. Early Child Development and Care.

[CR61] Rhoades B. L., Greenberg M. T., Domitrovich C. E. (2009). The contribution of inhibitory control to preschoolers’ social–emotional competence. Journal of Applied Developmental Psychology.

[CR62] Richard J. F., Schneider B. H. (2005). Assessing friendship motivation during preadolescence and early adolescence. The Journal of Early Adolescence.

[CR63] Rotheram-Fuller E., Kasari C., Chamberlain B., Locke J. (2010). Social involvement of children with autism spectrum disorders in elementary school classrooms. Journal of Child Psychology and Psychiatry.

[CR64] Rubia K., Russell T., Overmeyer S., Brammer M. J., Bullmore E. T., Sharma T., Taylor E. (2001). Mapping motor inhibition: Conjunctive brain activations across different versions of go/no-go and stop tasks. NeuroImage.

[CR65] Rutter M., Bailey A., Lord C. (2003). Social communication questionnaire (SCQ).

[CR66] Scheeren A. M., Banerjee R., Koot H. M., Begeer S. (2015). Self-presentation and the role of perspective taking and social motivation in autism spectrum disorder. Journal of Autism and Developmental Disorders.

[CR67] Sally D., Hill E. (2006). The development of interpersonal strategy: Autism, theory-of-mind, cooperation and fairness. Journal of Economic Psychology.

[CR68] Scheeren A. M., Begeer S., Banerjee R., Meerum Terwogt M., Koot H. M. (2010). Can you tell me something about yourself?: Self-presentation in children and adolescents with high functioning autism spectrum disorder in hypothetical and real life situations. Autism: The International Journal of Research and Practice.

[CR69] Scheeren A. M., de Rosnay M., Koot H. M., Begeer S. (2013). Rethinking theory of mind in high-functioning autism spectrum disorder. Journal of Child Psychology and Psychiatry.

[CR70] Schmitz, E. A., Banerjee, R., Pouw, L. B. C., Stockmann, L., & Rieffe, C. (2014). Better to be equal? Challenges to equality for cognitively able children with autism spectrum disorders in a social decision game. *Autism*, 1362361313516547. 10.1177/1362361313516547 24523411

[CR71] Sebastian C. L., Fontaine N. M. G., Bird G., Blakemore S.-J., De Brito S. A., McCrory E. J. P., Viding E. (2011). Neural processing associated with cognitive and affective theory of mind in adolescents and adults. Social Cognitive and Affective Neuroscience.

[CR72] Shaw A. (2013). Beyond “to Share or Not to Share”: The impartiality account of fairness. Current Directions in Psychological Science.

[CR73] Shaw A., Montinari N., Piovesan M., Olson K. R., Gino F., Norton M. I. (2014). Children develop a veil of fairness. Journal of Experimental Psychology: General.

[CR74] Sinclair, J. (1999). Why I dislike ‘‘person first’’ language. Retrieved September 24, 2012, from http://www.autcom.org/articles/defeated.html.

[CR75] Smith E. A. (2010). Communication and collective action: Language and the evolution of human cooperation. Evolution and Human Behavior.

[CR76] Sylwester K., Roberts G. (2010). Cooperators benefit through reputation-based partner choice in economic games. Biology Letters.

[CR77] Tager-Flusberg H. (2007). Evaluating the theory-of-mind hypothesis of autism. Current Directions in Psychological Science.

[CR78] Tanis M., Postmes T. (2005). A social identity approach to trust: Interpersonal perception, group membership and trusting behaviour. European Journal of Social Psychology.

[CR79] Tennie C., Frith U., Frith C. D. (2010). Reputation management in the age of the world-wide web. Trends in Cognitive Sciences.

[CR80] Thomae M., Zeitlyn D., Griffiths S. S., Van Vugt M. (2012). Intergroup contact and rice allocation via a modified dictator game in rural cameroon. Field Methods(San Diego, Calif.).

[CR81] Tomasello M., Melis A. P., Tennie C., Wyman E., Herrmann E. (2012). Two key steps in the evolution of human cooperation: the interdependence hypothesis. Current Anthropology.

[CR82] Tomasello M., Vaish A. (2013). Origins of human cooperation and morality. Annual Review of Psychology.

[CR83] Von Hippel W., Gonsalkorale K. (2005). ‘That is bloody revolting!’. Psychological Science.

[CR84] Wechsler D. (2011). Wechsler abbreviated scale of intelligence.

[CR85] White S., Hill E., Happé F., Frith U. (2009). Revisiting the strange stories: Revealing mentalizing impairments in autism. Child Development.

